# Autochthonous Transmission of *Coccidioides* in Animals, Washington, USA

**DOI:** 10.3201/eid2501.180411

**Published:** 2019-01

**Authors:** Allison E. James, Gabrielle Pastenkos, Daniel Bradway, Timothy Baszler

**Affiliations:** Washington State University, Pullman, Washington, USA

**Keywords:** *Coccidioides*, coccidioidomycosis, zoonotic infections, zoonotic diseases, mycoses, fungal diseases, fungal infections, veterinary medicine, zoonoses, Washington, fungi, United States

## Abstract

We report 5 cases of coccidioidomycosis in animals that were acquired within Washington, USA, and provide further evidence for the environmental endemicity of *Coccidioides immitis* within the state. Veterinarians should consider coccidioidomycosis in animals with compatible clinical signs that reside in, or have traveled to, south central Washington.

Since 2010, a total of 13 human cases of coccidioidomycosis have been reported in Washington, USA, that were acquired within the state (*1*). Soil samples from south central Washington were also found to harbor viable *Coccidioides immitis*, and whole-genome sequencing verified that the genotype obtained from soil matched that of the clinical isolate in 1 patient (*2**,**3*). This evidence strongly supports that the organism is endemic in Washington, well north of the previously defined geographic range. 

We undertook a thorough search of the Washington Animal Disease Diagnostic Laboratory (WADDL) database to identify all animal coccidioidomycosis cases in which autochthonous transmission was likely to occur. We confirm and report the 3 probable cases that were previously known to WADDL diagnosticians (*4*), as well as 2 newly identified cases. Of the 5 animal cases that have histories compatible with local transmission in Washington, 4 preceded known human cases.

## Case Reports

We searched the WADDL database for diagnoses of “coccidioidomycosis,” “mycosis, *Coccidioides* spp*.*,” and*“*osteomyelitis, coccidioidomycosis.” All records with owner addresses in Washington were further investigated for a history of travel. We identified 16 animal cases with a diagnosis of coccidioidomycosis since 1990 for which the animal’s primary residence was in Washington. We then excluded any cases in animals that had an unknown or unclear travel history or those that traveled outside of Washington, Oregon, Idaho, and Montana, regardless of the amount of time between travel and onset of disease. Thereafter, 5 animals remained that were highly likely to have been exposed to the disease within Washington.

The first case, in a 6-year-old female Labrador retriever, was reported in 1997. Five months before diagnosis, the dog developed a draining abscess caudal to the stifle that recurred after antimicrobial drug and steroid treatment. Subsequent skin biopsy and histopathology examination revealed a pyogranulomatous cellulitis. Fungal culture of the lesion ultimately identified *Coccidioides*, and the diagnosis was confirmed with a fungal DNA probe-hybridization assay (Mayo Clinic, Rochester, MN, USA). The dog resided in Seattle but had a history of only intrastate travel to Yakima, Washington, before the onset of disease. Travel history was obtained from the dog’s owner at the time of diagnosis and documented in the WADDL record (Figure, orange diamonds).

**Figure:**
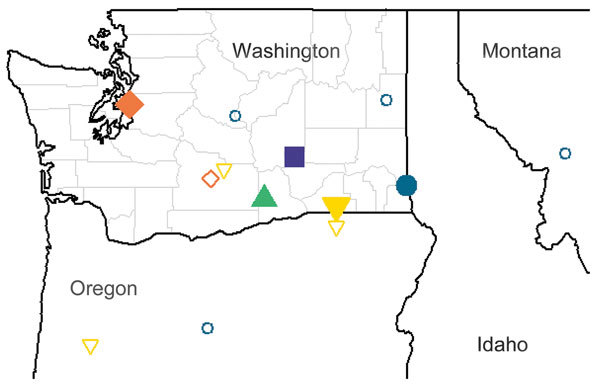
Locations of primary residence and travel of animals with apparently autochthonous coccidioidomycosis, Washington, USA. Five animals are depicted, each represented by a unique shape/color: orange diamonds, 6-year-old female Labrador retriever; blue circles, 14-year-old female quarter horse; green triangle, 6-year-old male German wirehaired pointer dog; purple square, 7-year-old female quarter horse; yellow inverted triangles, 2-year-old German shorthair pointer dog. Solid shapes depict the animal’s primary residence; hollow shapes indicate all travel locations of the 3 animals with local travel history.

The next case was in a 14-year-old female quarter horse that was brought to the Washington State University Veterinary Teaching Hospital (WSU–VTH) in 1999 for a painful swelling in the region of the withers that was unresponsive to antimicrobial drugs. Ultrasound of the lesion revealed a large fluid-filled mass that released purulent fluid when drained. An aspirate of the abscess was submitted for aerobic culture and revealed *Coccidioides* spp. According to documented conversations among WADDL diagnosticians, the owner, and the referring veterinarian, this mare lived in Clarkston, Washington, and had traveled out of state to Prineville and Rufus, Oregon, and Missoula, Montana. Intrastate travel history included Wenatchee and Spokane (Figure, blue circles).

In 2000, a 6-year-old male German wirehaired pointer dog was referred to WSU–VTH for a 4-week history of lameness, diffuse swelling of the right hindlimb, and a draining abscess near the right popliteal lymph node. The lesion had been unresponsive to debridement and antimicrobial drugs. WSU clinicians submitted tissue biopsies for histopathology examination, which revealed pyogranulomatous lymphadenitis and cellulitis. Subsequent fungal culture of the lymph node identified *Coccidioides*. PCR and sequencing using previously published methods confirmed the diagnosis, although this protocol cannot discriminate *C. immitis* from *C. posadasii* (*5*). This animal resided in Prosser, Washington, and was never taken outside the Yakima Valley. Travel history was reported by the owner and documented in WADDL records (Figure, green triangle).

A 7-year-old female quarter horse that developed progressively worsening paraparesis over the course of 2 weeks was identified in 2006 after admission to WSU–VTH. A mass over the thoracic spinal vertebrae was noted. Despite aggressive treatment with antimicrobial drugs, the animal’s condition rapidly deteriorated. The horse was euthanized; subsequent necropsy revealed a large, locally invasive paravertebral abscess that extended from T5 to T11. Histopathology examination revealed that the abscess was pyogranulomatous, with visible fungal spherules morphologically consistent with *Coccidioides* spp*.* The diagnosis was confirmed by DNA extraction from paraffin-embedded tissue blocks, followed by PCR and sequencing using methods that do not discriminate *Coccidioides* species (*5*). This horse lived in Othello, Washington, and had no out-of-state travel history according to the owner and documented in the WSU–VTH record (Figure, purple square).

The last case, a 2-year-old German shorthair pointer dog, was brought to WSU–VTH in late 2017 for persistent fever, weight loss, and severe progressive pneumonia that was refractory to multiple antimicrobial drugs. Histopathological findings from a right caudal lung lobectomy revealed severe pyogranulomatous pneumonia with rare fungal spherules. DNA was extracted from tissue samples for PCR and sequence analysis using published primers (*6*). In this case, sequencing confirmed the infection as *C. immitis* with 100% sequence identity to GenBank accession no. KF539899 and others and only 97.3% identity with *C*. *posadasii*. The primary residence for this dog was in College Place, Washington, although the dog frequently traveled to the Yakima area. The dog had occasionally traveled to Oregon, with multiple visits to Milton-Freewater and a visit to Eugene earlier in 2017 (Figure, yellow inverted triangles).

## Conclusions

In Washington, in contrast to other areas endemic for *Coccidioides* spp., animal cases are reported infrequently. Our analysis of the WADDL database has confirmed 5 cases highly likely to have been acquired in Washington, 4 of which were verified using molecular methods. Because we excluded animals with a history of travel to geographic areas traditionally endemic for coccidioidomycosis, regardless of time before clinical signs, it is possible that other animals in the WADDL database acquired the disease locally. Although unlikely, it should also be noted that for the 2 cases with a travel history to Oregon, out-of-state transmission cannot be dismissed.

As with humans, pulmonary disease is the most common clinical finding for coccidioidomycosis in animals, whereas bone and skin lesions are relatively uncommon (*7**,**8*). Of the 5 cases that we identified, 4 initially had extrapulmonary abscesses that were refractory to antimicrobial drugs. Except in rare instances, human and animal infections are acquired from inhalation of the highly infectious environmental form of *Coccidioides*, the arthroconidia (*9*). A few human cases have been reported after exposure to live or dead animals (*10**,**11*). In these cases, the spherule/endospore form has been implicated in human disease after exposure to infected tissues or fluids.

The Washington State Department of Health has worked to increase awareness of coccidioidomycosis with human and animal health providers by hosting community workshops and disseminating informational brochures (R. Wohrle, W. Clifford, D. Kangiser, Washington State Department of Health, pers. comm., 2018 Mar 7). Even so, it is likely that animal cases in Washington are being underdiagnosed and underreported. Canine serologic surveys would be valuable to determine the extent of exposure within the state and help to elucidate the geographic boundaries of the fungus in the region.
